# Do personal values and motivation affect women’s solo travel intentions in Taiwan?

**DOI:** 10.1057/s41599-022-01499-5

**Published:** 2023-01-04

**Authors:** Yi-Man Teng, Kun-Shan Wu, Ying-Chieh Lee

**Affiliations:** 1 School of Economics and Management, Yango University, 350015 Fuzhou, China; 2 Department of Business Administration, Tamkang University, Taipei, 25137 Taiwan

**Keywords:** Business and management, Business and management

## Abstract

Female solo travel is experiencing a global increase and specifically, gaining popularity in Asia. This study explores how personal values and female solo travel motivation affect travel behavior. Using a sample comprising 381 single females in Taiwan, partial least squares structural equation modeling was utilized to investigate the hypotheses. The results revealed Hypothesis 1 and Hypothesis 3 are supported, which verifies personal internal values significantly affect female solo travel motivation, and are identified as significant factors influencing female solo travel intention. Additionally, Hypothesis 5 is partially support, indicating the female solo travel motivations of escape/relaxation, relationship, and self-actualization contribute to the formation of positive female solo travel intention. As Hypothesis 2 and Hypothesis 4 are unsupported, external values have no impact on female solo travel motivation or any significant effect on female solo travel intention. This research adds to the vast gap in tourism literature by identifying the personal values and motivations of female solo travel, and benefits the development of the female solo travel market.

## Introduction

Although the COVID-19 pandemic influenced international travel decisions and changed tourism significantly during 2020 and 2021, French (2020) revealed the solo traveler market could be the first to return as the tourism sector recovers from the COVID-19 crisis. Furthermore, Wen et al. (2020) proposed that independent travel will likely increase after the COVID-19 pandemic. Solo travel is an increasingly common tourism option in modern society, even during the COVID-19 pandemic, which presents the travel industry with a great opportunity for recovery post-pandemic (Yang et al., 2022).

Owing to changes in social and political circumstances, the increasing interest from women to travel solo is evident in many parts of the world, and is a fast-growing segment of the travel industry (Wilson & Little, 2005; Dempsey, 2015). According to Solo Travel Statistics (2019), 72% of women in the USA prefer to travel alone and between 2015 and 2017, female solo bookings increased by 45%. Women currently account for almost two-thirds of travelers, particularly Asian women who occupy a growing proportion of contemporary tourism (Yang et al., 2017; Tan et al., 2018). The Klook poll (2019) also shows that in 2019, solo travel was more prevalent in Asia, 69% to 93%, compared to 60% to 69% in Western nations. Seow and Brown (2018) evidence Asian women have a growing interest in solo travel, and similarly, Bond (2019) states women traveling alone has become commonplace and can be attributed to travelers’ life changes.

Wilson and Little (2005) define female solo travelers as women traveling alone, without partners, family, or friends, who are in search of adventure, social interaction, education, and self-understanding, and are confident by themselves. McNamara and Prideaux (2010) consider female solo travelers to be women who travel to a destination alone and not as part of a group or organized tour. Gaining a life-changing experience, empowerment, liberation, identity, personal time, and freedom from domestic roles encourages women to consider traveling alone (Jordan & Gibson, 2005; Wilson & Harris, 2006). Similarly, Yang et al. (2018a) states female solo travel takes women out of their home environments and into unfamiliar destinations and cultures. In search of freedom, independence, empowerment, and autonomy, women traveling alone demonstrate a new style of travel.

Although there is support for the autonomy and freedom of female solo travelers, they still face danger and harassment, and encounter criticism and restrictions (Elliot, 2015). Women traveling alone also experience societal disapproval, unwanted attention, and sexual harassment (Karagöz et al., 2021). Furthermore, research evidences female solo travelers fear being attacked, abused, or harassed by men, which limits their use of the recreational spaces provided by their travel destination (Seow & Brown, 2018). Asian female solo backpackers encounter and overcome varied real and subjective risks (Wantono & McKercher, 2020). Yang et al. (2018b) argues that Asian female solo travelers respond to risk through a variety of gender-specific spatial and physical practices, which highlight tourist risk perception. They also discovered that female solo travelers’ experiences are more susceptible to social risks and psychological pressure.

Female solo travel has risen globally, drawing attention from academics and researchers. There is extensive literature focusing on the concept of perceived gender risks for female solo travelers such as assault, sexual harassment, and personal safety (Wilson & Little, 2005; Yang et al., 2018b; Kour & Gupta, 2019; Thomas & Mura, 2019; Kaba, 2021); travel experiences such as empowerment, surveillance, resistance, and self-discovery (Jordan & Gibson, 2005; Yang et al., 2019; Nikjoo et al., 2021); female solo travelers’ constraints (Nguyen, 2018; Schwab, 2019; Uatay et al., 2019; Ngwira et al., 2020; Bernard et al., 2022); psychological-social support (Karagöz et al., 2021); requirements and preferences of female solo travelers (Sebova et al., 2021); and motivations (Chiang & Jogaratnam, 2006; Seow & Brown, 2018; Breda et al., 2020; Terziyska, 2021).

There is still insufficient research focusing on the effect of personal values and motivations on female solo travel intention. Considering the importance of identifying value and motivations as key concepts in tourism consumption behavior (Hindley and Font, 2018), it is essential to identify the effect of personal values and motivations on female solo travel and the consequences thereof. In marketing and tourism research, personal values and motivations are important factors to better understand consumer behavior (Woosnam et al., 2016; Lin & Fu, 2016; Kim, 2020; Seow & Brown, 2018; Khan et al., 2019).

Female tourists are inhibited by vulnerability and their perceptions of potential risks, for example, walking alone at night or in isolated spaces (Brown et al., 2020). Asian female solo travelers also face other complications such as the influence of Confucianism, meaning they are often perceived as domesticated, dependent, vulnerable, and obedient (Yang et al., 2018a). Regardless of these perceptions and despite being bound by cultural beliefs and stricter social expectations, more Asian women are breaking stereotypes by preferring to travel alone (Yang et al., 2017). In Taiwan, the rise in female solo travel has been greatly influenced by factors such as globalization, economic expansion, and democratic tendencies. In 2019, there were 8,736,907 (51.09%) Taiwanese female outbound travelers, which is higher than the number of males (Taiwan Tourism Bureau, 2020). As Taiwanese women become an extensive part of Asian tourists and a significant demographic for outbound tourism, it is crucial the motivations for Taiwanese female solo travelers are studied. Existing relevant studies do not explore this specific demographic in detail (Su & Wu., 2020). Thus, the focus of this study is female solo travel intention of women in Taiwan, from the perspective of personal values, motivations, and travel experience. This research aims to show a holistic perspective of female solo travel intention, and explores how personal values and female solo travel motivation affects travel behavior.

The remainder of this study is structured as follows: “the Literature Review and Hypothesis Development” section includes reviews of relevant literature and hypothesis development; the “Research method” section provides sample details, measurements of constructs, and data analysis; the “Results” section explains the sample profile, the exploratory factor analysis (EFA) results, confirmatory factor analysis (CFA) results, and the path coefficient of the structural model; the “Discussion” sections offers a discussion of the empirical results, theoretical and practical implications, limitations and protentional future research suggestions.

## Literature review and hypothesis development

### Personal values

According to Schwartz (1992), the definition of personal values is “the transcending motivating life goals and guiding principles of an individual’s life”. Within the tourism industry, personal values are linked to tourism behavior, including tourists’ decision-making processes, motivations, and activity preference (Lin & Fu, 2016). From a theoretical perspective, it is significant to compare the explanatory power of an individual’s personal values on travel behavior. Personal values also provide accurate clarification for travel behavior, as individuals with different personal values exhibit different travel behavior patterns (Mehmetoglu et al., 2010). Li and Cai (2012) and Kim’s (2020) empirical tourism literatures support the above implications. Academics and practitioners can gain valuable information by exploring the nexus between personal values and travel behavior.

List of values (LOV) (Kahle et al., 1986) is the most extensively applied values scale in tourism and leisure consumer studies (Muller, 1991; Madrigal & Kahle, 1994; Chen & Sasias, 2014; Li, Cai, & Qiu, 2016; Lindberg et al., 2019; Wen & Huang, 2019; Li & Cai, 2012; Mauri & Nava, 2021). There are nine terminal values in the LOV: self-fulfillment, self-respect, sense of accomplishment, security, sense of belonging, warm relationships with others, well-respected, fun and enjoyment, and excitement. The nine values are classified into two categories: external and internal values (Li & Cai, 2012). LOV is the prevalent tool applied in value studies and has been used previously to investigate tourist and traveler behavior. Based on the suggestions of Ladhari et al. (2011), LOV is simpler, more effectively managed and has greater predictive utility in consumer behavior than Values and Lifestyles (Mitchell, 1983), and the Rokeach Value Survey (Rokeach, 1973). In line with rising interest in personal values and awareness of current tourism literature, this research employs LOV to investigate personal values as the antecedent variables effecting female solo travel motivation and solo travel intention.

### Solo travel motivation

Motivation is the predominant catalyst for humans to complete action, move forward, and realize their goals. (Luvsandavaajav & Narantuya, 2021). Hsu et al. (2017) state that when an individual’s need is stimulated, motivation will emerge to guide people to take the required action to satisfy their needs. Simply put, motivation is the tendency to fulfill an individual’s psychological needs (Bromley, 1990). Travel motivation is one of the most important contributors toward travelers’ decision-making behavior, thus gaining a greater comprehension of travelers’ motivation is crucial to establishing tourists’ behavioral patterns (Luvsandavaajav & Narantuya, 2021).

In tourism academicians, tourists’ motivation to go to a specific destination and the reason they choose to travel are both topics of debate (Keshavarzian & Wu, 2017; Wong et al., 2017; Pereira et al., 2022; Katsikari et al., 2020; Luvsandavaajav & Narantuya, 2021). Travel motivation causes individuals to participate in tourist activities and compels travelers to take the required actions to satisfy their needs (Fodness, 1994; Pizam & Mansfeld (1999)). Yoon and Uysal (2005) regard travel motivation as an internal power that stimulates and inspires people to choose a specific destination for the purpose of obtaining expected benefits and satisfaction. Mayo and Jarvis (1981) pointed out travelers are driven by psychological elements, and travelers’ behavior is accounted for by motivation.

The cultural and biological force that provides direction and value to travel patterns, decisions, experiences, and behaviors is commonly referred to as tourism motivation (Pearce, 2005). As tourists are from different countries and cultures, and have differing characteristics and tourist product preferences, travel motivation has a heterogeneous structure (Çelik & Dedeoğlu, 2019; Kozak, 2002). Owing to this heterogeneous structure, there is no global theory that can prove travelers’ motivation. Thus, diverse theories have been researched and developed accordingly (Robinson et al., 2011).

Yoon and Uysal (2005) show that anthropology interprets motivation as moving away from a routine environment to seek authentic experiences, while psychology explains motivation via emotional and cognitive motives, or internal and external motives. Travel motivation is a combination of requirements and attitudes that compel an individual to join in touristic activities (Pizam et al.,^1979^).

Travel Career Ladder was developed based on Maslow’s hierarchy of needs theory, which demonstrates people tend to change their travel motivation based on relationships, stimulation, self-esteem and development, and fulfillment of their travel career ladder (Pearce,^ 1988^; Pearce & Lee, 2005). The Push-Pull Model states that travel decisions are motived by push factors and pull factors in a two-stage process (Uysal et al., 2008). Destination features and external motivation are regarded as the pull factors, and internal motivation belongs to the push factors (willingness and needs etc.) of an individual. Both of which are present during the decision-making process.

As women travel alone for a variety of reasons, solo travel motivation is still changeable; however, some studies specifically investigate the motivation for female solo travel. For example, Chiang and Jogaratnam (2006) identify the four motivations for female solo travel as experience, escape, relaxation, and socialization. Yang et al. (2019) state the reason for Asian female solo travel is self-discovery, which is constructed by challenging the social expectations for Asian women. Some research shows that female solo travelers, in their search of freedom, embrace the autonomy, independence, and empowerment gained by traveling alone (Yang et al., 2018a, 2018b). Thus, the intrinsic motivations for female solo travelers are their need to feel challenged, empowered, and autonomous (Bianchi, 2016; Wilson & Little, 2005).

Other research identifies the motivations for most women who travel alone are a need to find themselves, not having a travel companion, freedom of choice, experience and adventure, and to escape from daily routines (Breda et al., 2020). Similarly, women choose solo travel for adventure, independence, personal fulfillment, individuality and escape (Pereira & Silva, 2018). Female solo travelers desire an escape from their daily lives and look for active resistance against the gender stereotypes they are used to. Through solo travel, women can reconfigure their identity, and at the same time, change the power relationship that maintains the mainstream social concept.

Existing literature suggests cultural influences and constraints effect female solo travel motivation. Western female travelers from individualist cultures are often characterized as independent and therefore, presumed more likely to embark on solo travel (Yang et al., 2019). Research has identified that for women from advanced countries, the key motivations for solo travel in Australia are self-actualization and self-construal (Yang et al., 2022). Confronting stereotypes, the motivations of British, Australian, and American female solo travelers are feelings of freedom, autonomy and empowerment, confirmation of their identity, and increased self-esteem (Bianchi, 2016).

Although female solo travelers share some commonalities, the interpretation of tourism in developing Muslim countries differs from advanced western countries (Cohen & Cohen, 2015). Islam is a religion that permeates many facets of Muslim life, particularly among women and their leisure activities (Moghadam et al., 2009). In patriarchal Muslim communities, hegemonic masculinity has degraded the role of women (Hosseini et al., 2022). Therefore, women face various restrictions, such as not being allowed to travel alone or requiring permission from their father or husband to travel (Seyfi et al., 2022). The severe domination of Islamic laws on cultures in traditional Muslim countries has weakened the effect of female sole travel motivation. However, recent study results indicate that solo travel motivations are independence, self-empowerment, freedom and flexibility, and exploration (Hosseini et al., 2022). The discussed literature suggests the differences between the female solo travel motivations of women in developed and developing countries is not that dissimilar.

### Hypotheses development

Homer and Kahle (1988), and Hofstede and Hofstede (2005) argue that values are the basis of culture and humanity, therefore, human behavior could be predicted and indicated by values. During the motivation development process, values are the guidelines when replying to stimulus (Kahle, 1983), and help to assess the tourism environment and destinations of the objects or events. In the perspective of cultural and social factors, values impact an individual’s motivation and are identified as the external environment signals. Previous studies identify two dimensions of values: external and internal (Li & Cai, 2012).

Several studies argue personal values significantly impact an individual’s behavior. Ateljevic (1997) posits that values obtained in daily life are dedicated to the making of motivations, and simultaneously studied the influence of value systems on tourism motivation in order to determine how a situational influence represented by values affects tourists’ motivation. Furthermore, Li and Cai (2012) empirically tested the impacts of personal values (internal and external values) on motivations and behavioral intention and found they had a direct impact on travel motivation. Woosnam et al. (2016) argue that tourism literature should investigate the connection between values and motivations on the attendance levels of particular tourist attractions. Their research confirmed that values significantly predict the motivations and potential attendees of the Winnipeg Fringe Theater Festival.

Tourists consider the nexus between personal values and the quality of the leisure and travel activities, and the connection between values and actual tourism behavior important (Pitts & Woodside, 1986). In a study on tourism, Hindley and Font (2018) point out that values and motivations have a complex interrelationship, and argue that values are the underlying psychological determinants of consumers’ purchase intentions, thus stimulating ethical consumption. In line with prior research results, Hede et al. (2004) demonstrate a connection between personal values, satisfaction, and behavioral intentions of participants in urban hallmark events. More recently, young travelers’ self-transcendence values contain predictive power over motivations and behavioral intention for sustainable tourism among young travelers (Cavagnaro et al., 2021).

Travel motivation is essential to predict travel behavioral intention (Jang et al., 2009; Li & Cai 2012; Khan et al., 2019; Hosany et al., 2020;), thus many studies focus on the nexus between travel motivation and tourists’ behavioral intention. Yoon and Uysal (2005) reveal tourist motivations are antecedents for tourist satisfaction and tourists’ behavioral intention, particularly with regard to re-visiting and recommendations to others. Jang and Feng (2007) state that the motivation for seeking new experiences significantly affects tourists’ intentions to revisit the destination within a three-year period. A study on tourists in France shows that travel motivation significantly positively effects behavioral intention (Prayag, 2012). Li and Cai (2012) empirically test the impacts of travel motivation on behavioral intention, and evidence that the travel motivation of Novelty and Knowledge significantly positively impacts behavioral intention.

Khuong and Ha (2014) state that push motivations and pull motivations have a positive correlation between travelers’ satisfaction and behavioral intention. Luvsandavaajav and Narantuya (2021) apply travel push motivations and pull motivations to examine and confirm the correlation between values, perceived benefits, and behavioral intention. Their findings suggest travel motivation (push and pull factors) are significant constructs of behavioral intention. Furthermore, travel motivation as internal sociopsychological drivers, such as novelty-seeking, escape-seeking, assurance-seeking, and interaction-seeking motivations, can influence travel decision formation (Maghrifani et al., 2022). Based on this discussion, Hypotheses 1 to 5 (H1-H5) are:

H1: Internal values positively impact on travel motivation.

H2: External values positively impact on travel motivation.

H3: Internal values positively impact on behavioral intention.

H4: External values positively impact on behavioral intention.

H5: Travel motivation positively impact on behavioral intention.

**Fig. 1:**
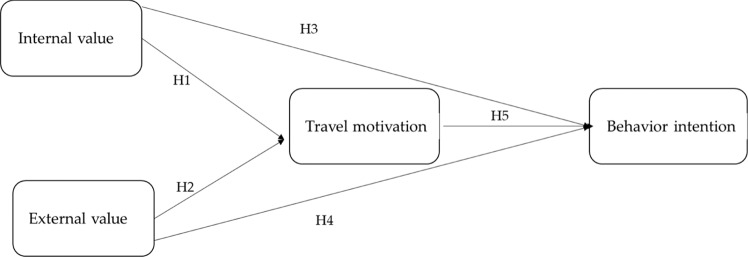
Female solo travel intention research model. The model examines the relationship between internal values, external values, travel motivation and behavioral intention.

Figure 1 illustrates the conceptual model.

## Research methods

### Sample

A quantitative approach based on structured self-administered questionnaires was used to both assess the conceptual model and test the proposed hypotheses. The snowball sampling method was used to collect data from single females in Taiwan. An online survey was distributed via Line using a Google Forms link. Before the questionnaire was distributed to the participants, they were told the purpose of the survey and once they had agreed to participate, were provided with the questionnaire to complete. In determining the sample size, the rule-of-thumb recommendations from Sekaran and Bougie (2010) were followed. The sample size should be greater than 30 and less than 500, and several times (preferably more than 10 times) the number of variables in multivariable studies. Based on this consideration and the rule of thumb, the minimum sample size of this study was greater than 35 (35*10) or equal to 350.

### Measurements of constructs

The questionnaire comprised four sections: (1) Demographics, including participants’ age, education, and average annual income; (2) Personal Values; (3) Solo Travel Motivation; and (4) Solo Travel Intention. The items in the survey questionnaire were adapted from prior studies (Table 1) and measured using a five-point Likert-type scale (1 = strongly disagree to 5 = strongly agree).

**Table 1:** Constructs and measurement items.

Constructs/questionnaire items
Internal values (IV)
IV1. Sense of self-fulfillment.
IV2. Excitement.
IV3. Sense of accomplishment.
IV4. Fun and enjoyment in life.
IV5. Warm relationships with others.
External values (EV)
EV1. Self-respect.
EV2. Being well-respected.
EV3. Sense of security.
EV4. Sense of belonging.
Female solo travel motivation (MO)
MO 1. Find active, adventurous, exciting things to do.
MO 2. Show-off.
MO 3. Share travel experience with others.
MO 4. Meet new people.
MO 5. Mix with fellow travelers.
MO 6. Escape other places/pressures.
MO 7. Relax and take it easy.
MO 8. Escape from routine.
MO 9. Overcoming challenges.
MO 10. Seeking intellectual enrichment.
MO 11. Learn something new.
MO 12. Because it is a talked about, well-publicized destination.
MO 13. Special food.
MO 14. Comfortable lodging.
MO 15. Fulfill a lifelong dream and ambition.
Female solo travel intention (IN)
IN 1. I think it worthwhile to travel alone.
IN 2. I would travel alone if my budget allowed.
IN 3. Compared with traveling with others, I prefer to travel alone.

Personal values were measured using a nine-item LOV scale (Kahle, 1983). Travel motivation was measured using Pereira and Silva’s (2018) fifteen-item scale based on Loker-Murphy’s (1997) modified Travel Career Patterns theory (Pearce, 1988). There are four items for Escape/Relaxation, five for Relationship, three for Self-actualization/Development, and three items for Fulfillment. Three items from Reisinger and Mavondo (2005), and Lam and Hsu’s (2006) scale were extracted to measure female solo travel intention. An example of one of these items is: “I think traveling alone is worth it.”

### Data analysis

SPSS 26.0 and VISUAL PLS 1.04b were utilized to descriptive statistics analysis, Harman’s single-factor test, and assess the structural data. To check for any common method variance issues, Harman’s single-factor test was used. EFA was conducted on female solo travel motivation and personal values to examine the dimensionalities and psychometric properties. In the second stage, the associations among personal values, female solo travel motivation, and solo travel intention were empirically tested using the structural equation modeling (SEM) technique. The research used SEM with partial least square (PLS) for model estimation and hypothesis testing. Social science studies prefer applying PLS-SEM for multivariate analysis (Abid et al., 2020; Pan et al., 2021).

## Results

### Descriptive statistics

The survey received 409 responses in total; however, 28 invalid questionnaires were removed, leaving 381 valid questionnaires. The effective sample recovery rate is 93.2%. Table 2 shows the respondents’ basic demographic information. A descriptive analysis of all variables was performed to assess their normality prior to testing the econometric and structural models. The results show the sample skewness is between −0.057 and 0.985, and the kurtosis risk is between −1.269 and 0.816, which does not violate the normality hypothesis.

**Table 2:** Summary of descriptive statistics.

Item	Classification	Frequency	Percentage (%)
Age	30–34 years old	106	27.8
	35–40 years old	71	18.6
	41–45 years old	84	22.0
	46–49 years old	50	13.2
	≧ 50 years old	70	18.4
Education level	College diploma or lower	89	23.4
	Bachelor	188	49.3
	Master’s or higher	104	27.3
Average annual income (NT dollars)	500,000 or below	100	26.2
	500,001–1,000,000	174	45.7
	1,000,001–1,500,000	70	18.4
	1,500,001–2,000,000	21	5.5
	2,000,001 or above	16	4.2

### Common method bias and multicollinearity evaluation

This study tested the common method bias (CMB) using several methods, including Harman’s one-factor test. The variance explained by the first factor loading is only 25.047% of the total variance, which is less than 50%, as suggested by Podsakoff and Organ (1986). This means CMB is not obvious in the dataset. Furthermore, the results of the full variance inflation factors (VIF) can be utilized to assess CMB and allows for a more conservative test than traditional EFA (Kock, 2013). The VIF should be less than 3.3 to exclude CMB (Kock & Lynn, 2012). In the model, VIF values are less than five, as stated in recent articles (Farooq et al., 2022; Talwar et al., 2020). Therefore, the data did not identify any multicollinearity issues within the constructs (Hair et al., 2020). As the CMB was tested using the mentioned-above different methods, it can be assumed that CMB is not an issue.

### Exploratory factor analysis (EFA)

The EFA procedure helps to reduce the multicollinearity or correlation of error terms among indicators in CFA (Li & Cai, 2012), thus EFA is necessary in this study.

Regarding the construction of the personal values, two factors were extracted that were capable of explaining 59.471% of the variance in the variables (Table 3). Those two factors were labeled Internal Values and External Values. This result is similar to those of previous studies (Li & Cai, 2012), except the statement “fun and enjoyment in life” is included in Internal Values, and the statement “being well-respected” is included in External Values. The reliability test shows the factors are higher than 0.7.

**Table 3:** EFA of personal values.

Factor/item	Loading		Eigenvalue	Variance explained (%)	Corrected item-total correlation	Cronbach’s *α*
**Factor 1: Internal values**			2.703	30.033	0.661	0.792
Sense of self-fulfillment		0.791			0.506	
Excitement		0.760			0.634	
Sense of accomplishment		0.723			0.634	
Fun and enjoyment in life		0.641			0.540	
Warm relationships with others		0.599			0.543	
**Factor 2: External values**			2.649	29.438		0.797
Self-respect		0.805			0.630	
Being well-respected		0.777			0.662	
Sense of security		0.758			0.569	
Sense of belonging		0.678			0.579	
Total				59.471		0.844

In terms of the construct of female solo travel motivation, 15 items are retained to generate a five-factor solution, explaining 61.761% of the total variance. Furthermore, five items load highly on Factor 1: Relationship; four items load highly on Factor 2: Escape/Relaxation; three items load highly on Factor 3: Self-actualization/Development; and three items load highly on Factor 4: Fulfillment. The reliability coefficients range from 0.632 to 0.803, which indicates satisfactory levels of internal consistency (Table 4).

**Table 4:** EFA of female solo travel motivation.

Factor/item	Loading	Eigenvalue	Variance explained (%)	corrected item-total cor relation	Cronbach’s *α*
**Factor 1: Relationship**		3.019	20.127		0.805
Find active, adventurous, exciting things to do	0.831			0.693	
Show-off	0.779			0.663	
Share travel experience with others	0.749			0.649	
Meet new people	0.659			0.513	
Mix with fellow travelers	0.556			0.466	
**Factor 2: Escape/relaxation**		2.329	15.525		0.750
Escape other places/pressures	0.826			0.581	
Relax and take it easy	0.726			0.570	
Escape from routine	0.672			0.561	
Overcoming challenges	0.574			0.472	
**Factor 3: Self-actualization/development**		2.136	14.242		0.723
Seeking intellectual enrichment	0.864			0.657	
Learn something new	0.816			0.575	
Because it is a talked about, well-publicized destination	0.595			0.416	
**Factor 4: Fulfillment**		1.780	11.866		0.632
Special food	0.794			0.484	
Comfortable lodging	0.749			0.505	
Fulfill a lifelong dream and ambition	0.566			0.344	
Total			61.761		0.826

### Assessment of the measurement model

CFA was utilized to validate the proposed factor structure and confirm whether modification is required. As proposed by Anderson and Gerbing (1988), a two-step CFA was conducted to evaluate each construct separately and assess the overall measurement model.

First, the individual reliability of each item is determined by analyzing the simple loadings or correlations of the measures or indicators with their respective construct. To indicate a good fit, the indicators’ external loadings must be higher than 0.7 (Hair et al., 2014). The item “warm relationships with others” in Personal Values, was deleted due to the low standardized factor loading, as suggested by Hair et al. (2014). For Female Solo Travel Motivation, the items “mix with fellow travelers”, “because it is a talked about, well-publicized destination”, and “fulfill a lifelong dream and ambition” were also removed due to the low standardized factor loading (Hair et al., 2014).

Second, the Cronbach’s alpha and CR values exceed the minimum requirement of 0.7, suggesting that reliability is satisfactory (Table 5) (Nunnally & Bernstein, 1994; Hair et al. (2017, 2020); Yusof et al., 2012). Third, the AVE values are more than the threshold value of 0.50 (Nunnally & Bernstein, 1994; Hair et al. (2017, 2020); Yusof et al., 2012), meaning the convergent validity is satisfactory.

**Table 5:** Confirmatory factor analysis (CFA).

Variable	Items	Mean	Std	Loading	*t*-value	CR	AVE	Cronbach’s *α*
Internal value	Sense of self-fulfillment	4.244	0.641	0.784	25.237	0.850	0.586	0.761
	Sense of accomplishment	4.241	0.668	0.757	24.133			
	Fun and enjoyment in life	4.381	0.661	0.741	22.489			
	Excitement	3.808	0.816	0.781	32.735			
External value	Self-respect	4.094	0.701	0.750	21.151	0.866	0.618	0.797
	Being well-respected	4.346	0.645	0.835	35.864			
	Sense of security	4.486	0.610	0.743	16.693			
	Sense of belonging	4.168	0.687	0.812	24.615			
Relationship	Find active, adventurous, exciting things to do	4.189	0.755	0.824	42.151	0.876	0.639	0.811
	Show-off	4.425	0.655	0.823	36.700			
	Share travel experience with others	4.262	0.598	0.750	23.996			
	Meet new people	4.420	0.630	0.800	30.860			
Escape/relaxation	Escape other places/pressures	3.743	0.841	0.765	26.128	0.842	0.572	0.750
	Escape routine	3.472	0.803	0.755	19.950			
	Relax and take it easy	4.071	0.745	0.798	40.008			
	Overcoming challenges	3.522	0.826	0.704	21.490			
Self-actualization/development	Learn something new	3.016	1.093	0.899	42.135	0.898	0.815	0.773
	Pursue a hobby or interest	3.094	1.060	0.907	38.471			
Fulfillment	Special food	3.869	0.873	0.937	39.513	0.842	0.730	0.656
	Comfortable lodging	3.433	0.905	0.762	11.264			
Female solo travel intention	I think it worthwhile to travel alone	3.861	0.931	0.915	98.230	0.936	0.831	0.897
	I would travel alone if my budget allowed	3.596	1.061	0.939	157.329			
	Compared with traveling with others, I prefer to travel alone	3.094	1.045	0.880	65.291			

Discriminant validity is assessed using the correlation between variables and constructs, and by comparing the square root of AVE values with the correlations between constructs (Fornell & Larcker, 1981). The results of examining the constructs indicate the discriminant validity is satisfactory (Table 6).

**Table 6:** Fornell–Larcker criterion analysis of the model.

	(1)	(2)	(3)	(4)	(5)	(6)	(7)
(1) Internal	**0.766**						
(2) External	0.493**	**0.786**					
(3) Relationship	0.478**	0.261**	**0.800**				
(4) Escape/relaxation	0.440**	0.145**	0.408**	**0.756**			
(5) Self-actualization/development	0.104*	0.043	0.146**	0.227**	**0.903**		
(6) Fulfillment	0.208**	0.239**	0.310**	0.396**	0.307**	**0.854**	
(7) Female solo travel intention	0.351**	0.003	0.377**	0.432**	0.310**	0.180**	**0.912**
Mean	4.1686	4.2736	4.3241	3.7021	3.0551	3.6509	3.5171
Standard deviation	0.5343	0.5212	0.5287	0.6079	0.9718	0.7670	0.9231

Note: Values in bold represent the square root of AVE. Note: ** denotes p < 0.01.

### Hypothesis testing procedure and results

The PLS method is used to test the hypotheses as it focuses on interpreting path coefficients and variances, rather than overall model fit (Pavlou & Fygenson, 2006). The advantage of this method is that the assumption of normal distribution required by structural equation models is relaxed, meaning more complex models can be estimated using smaller sample sizes.

The empirical results evidence that internal values have a significant positive effect on the motivations of Escape/Relaxation (*β* = 0.495, *t* = 10.304, *p* < 0.01), Relationship (*β* = 0.467, *t* = 9.822, *p* < 0.01), Self-actualization/Development (*β* = 0.111, *t* = 1.776, *p* < 0.05), and Fulfillment (*β* = 0.337, *t* = 5.303, *p* < 0.01) (Table 7). Thus, H1-1 to H1-4 are supported. However, external values have no significant influence on motivational factors, thus H2-1 to H2-4 are not supported. In addition, internal values significantly positively influence female solo travel intention (*β* = 0.239, *t* = 3.802, *p* < 0.01), whereas external values significantly negatively affect female solo travel intention (*β* = −0.200, *t* = −3.694, *p* < 0.01). Thus, H3 is supported, while H4 is not supported.

**Table 7:** Path analysis of structural model.

Hypothesis	Path coefficient	Std. error	*t*-value	Consequence
H1-1: Internal values → Escape/relaxation	0.495**	0.048	10.304	Supported
H1-2: Internal values → Relationship	0.467**	0.048	9.822	Supported
H1-3: Internal values → Self-actualization/development	0.111*	0.063	1.776	Supported
H1-4: Internal values → Fulfillment	0.337**	0.064	5.303	Supported
H2-1: External values → Escape/relaxation	−0.082	0.055	−1.506	Not supported
H2-2: External values → Relationship	0.041	0.033	1.240	Not supported
H2-3: External values → Self-actualization/development	−0.008	0.045	−0.178	Not supported
H2-4: External values → Fulfillment	0.061	0.052	1.165	Not supported
H3: Internal values → Solo travel intention	0.239**	0.063	3.802	Supported
H4: External values → Solo travel intention	−0.200**	0.054	−3.694	Not supported
H5-1: Escape/Relaxation → Solo Travel Intention	0.237**	0.059	4.037	Supported
H5-2: Relationship → Solo Travel Intention	0.196**	0.052	3.779	Supported
H5-3: Self-actualization/development → Solo travel intention	0.216**	0.044	4.941	Supported
H5-4: Fulfillment → Solo travel intention	−0.015	0.040	−0.377	Not supported

*denotes *p* < 0.05; **denotes *p* < 0.01.

**Fig. 2:**
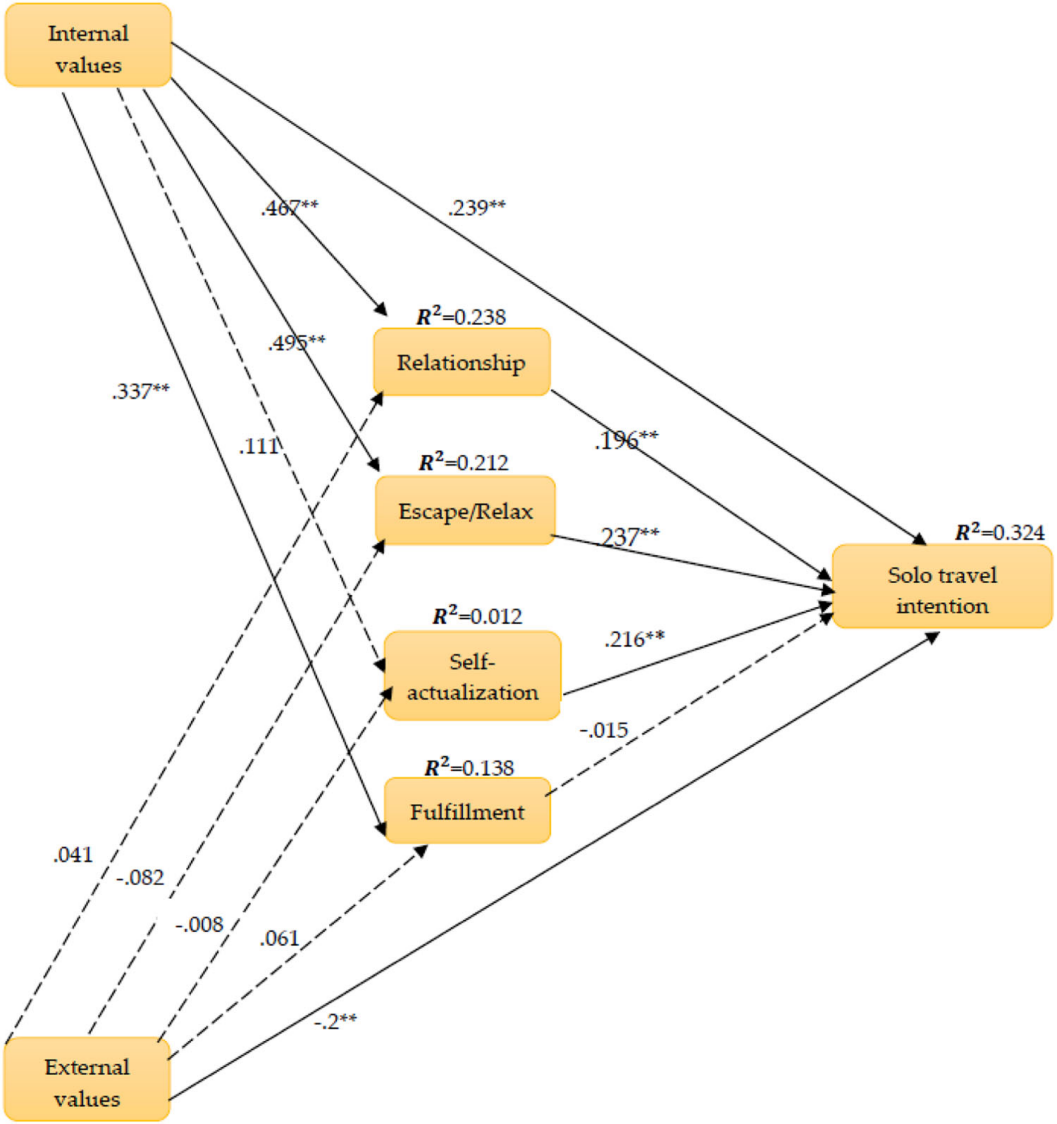
Path diagram of the structural model (**denotes *p* < 0.01). Internal values have a significant relationship with travel motivation and solo travel intention. Subsequently, travel motivation had a partially supported effect on solo travel intention.

Furthermore, the results also evidence that the motivations of Escape/Relaxation (*β* = 0.237, *t* = 4.037, *p* < 0.01), Relationship (*β* = 0.196, *t* = 3.779, *p* < 0.01), and Self-actualization/Development (*β* = 0.216, *t* = 4.941, *p* < 0.01) have a significant positive influence on female solo travel intention, but Fulfillment has no impact on female solo travel intention (*β* = −0.015, *t* = −0.377, *p* > 0.05). Thus, H5-1, H5-2, and H5-3 are supported, whereas H5-4 is not supported. The path graph of the proposed model is presented in Fig. 2.

## Discussion

The number of women embracing the autonomy of traveling independently is growing. Searching for freedom, independence, and empowerment, female solo travelers choose unfrequented or ‘off the beaten track’ destinations (Wilson & Little, 2005). Research focusing on the effect of personal values and motivations on female solo travel intention remains insufficient, and has long been neglected in female solo travel literature. This study aims to identify the personal values and motivations that support the increase in female solo tourist behavior to fulfill the current research deficiency in tourism literature. The results of this research both verify the findings of previous studies and elicit new information.

### Theoretical implications

Although solo travel is one of the fastest growing areas of the tourism industry, research is still limited, particularly in understanding what motivates female travelers’ desire to travel alone. This study and its findings contribute to existing tourism literature in several ways.

First, based on the research sample, results confirm that personal internal values significantly effect female solo travel motivation. Direct influences from personal internal values and female solo travel intention are also identified. This finding is consistent with the contentions of Woosnam et al. (2016) and Cavagnaro et al. (2021), who evidence travelers’ values have a predictive effect on motivations and behavioral intention.

Females who have internal values tend to form positive female solo travel motivation and intention toward a solo trip, which aligns with previous studies that evidence females’ emotionality influences their ability to manage situations (Costa et al., 2017). The internal values of sense of self-fulfillment, accomplishment, fun and enjoyment in life, and excitement have a similar influence as emotionality does on female solo travelers’ motivation and intention. This finding concurs with Li et al.’s (2016) propositions, who indicate that internal values have significant effects on tourist behavioral intention.

A female’s internal values enable them to participate in the process of women’s freedom and empowerment, which supports the findings of prior studies that through female solo travel, women can transcend the system and societal roles, stereotypical traditions, and social expectations (Seow & Brown, 2018; Bernard et al., 2022). Overall, this study evidences that personal internal values influence female solo travel motivation, with escape/relaxation being the primary contributor.

Second, the results also confirm that external values have no impact on female solo travel intention or any significant effect on female solo travel motivation. The reason for this could be that the construct of female solo travel motivation in this research model comprises the subjective emotions of self-respect, being well-respected, sense of security, and sense of belonging, which are related to emotionally dominant internal values. Hence, predicting the variation effect on the selected sample’s travel motivation and travel intention is arduous when investigating object-directed external values (Prentice,^1987^; Li et al., 2016).

Third, the empirical results confirm the significant effects of solo travel motivation on female solo travel intention. This finding concurs with Hosany et al. (2020). The motivations for female solo travel in this study are related to seeking escape, relaxation, relationships (meeting new people), and self-actualization/development. These motivations influence female solo travel intention in the future, particularly escape/relaxation as this is the most prominent factor affecting female solo travel motivation. Therefore, escaping from daily duties and life pressures, enjoying freedom, and reflecting on their own lives all have a transformative impact on the intention of women to travel alone.

### Managerial/social implications

This study’s findings have crucial implications for the hospitality and tourism industry and its managers. First, the results identify the role of personal values in determining female solo travel behavior. Tourism practitioners and destination marketers should appeal to the primary female internal values of sense of self-fulfillment and accomplishment, fun and enjoyment in life, and excitement to improve the overall travel package and destination features. For example, when proposing a luxury tour, it could be marketed as a spiritual journey to fulfill the pursuit of well-being, self-fulfillment, and accomplishment, and thus ultimately increase the desire for females to travel alone. When proposing an ultimate tour, it could be offered as an in-depth cultural and informative journey that satisfies the personal internal values of fun and accomplishment, conforming to the desires of the female solo traveler.

Second, as identifying female solo travel motivation is significant to determining female solo travel intention, it is suggested the tourist industry markets specifically to this demographic and provides special travel itineraries, packages, tourism products, and attractions aimed at fulfilling the female solo traveler’s desire to escape and/or relax. Traveling that achieves the pursued attributes will assist in the development of a positive attitude and intentions toward female solo travel.

Third, it is also suggested that travel enterprises evaluate the personal values of female travelers according to the LOV (Kahle & Kennedy, 1988) to understand individual travel motivations, so as to improve the willingness of women to travel alone. Enterprises should aim to meet the needs of female solo travelers by focusing their advertising strategies on this niche market. Such a strategy could more accurately develop the tourism products required to target the female solo traveler market, and ultimately improve the enterprise’s position and enhance their brand loyalty. Finally, providing tourism marketers with recommendations for development and service enhancement of female solo travel products would be beneficial as this is a fast-growing and lucrative market.

### Limitations and future research

Although this study identifies the effect of personal values on female solo travelers’ behavior as well as the influence on female solo travelers’ motivation, it still has some limitations. First, the research sample uses the snowball sampling method to collect data from single females in Taiwan via Line. The generalizability of the study findings is limited as it only presents the viewpoints and personal values of females in Taiwan. The results cannot be generalized for females from different countries and cultures as a whole, thus future research should investigate more diverse countries and cultures.

Tims et al. (2013) insists only longitudinal research completes the path in the theoretical model, thus the second limitation is the possibility of longitudinal parameters. Third, travel motivation will change dynamically over time, depending on travel experience behaviors. It would be interesting to study past travel experiences as a construct in future research models.

Fourth, several studies raise the issue of safety and security, and suggest greater protection for female solo travelers against male leering and sexual violence (Berdychevsky & Carr, 2022; Su & Wu, 2020). Future studies should address legal resources, facilities, and policies to promote female solo travel, such as female-only public spaces, subway cars and railways, and the need for female-only floors in hotels and other accommodation.

Fifth, tourist behavior and assessments are significantly influenced by emotions and cognitive processes (Hosany et al., 2021). However, the current literature ignores the emotional and cognitive implications of tourist behavior (Lee & Lee, 2021). Individual tourists may evaluate the same event differently cognitively and emotionally. Considering this viewpoint, this study encourages future research on female solo travel to examine cognitive and emotional consequences, in order to fill in the gaps in this area. Lastly, future studies should specify whether women are traveling alone domestically or internationally, as this alters the type of limitations that apply.

## Data Availability

The datasets generated during and/or analyzed during the current study are available from the corresponding author on reasonable request.
